# What Is Direct Allorecognition?

**DOI:** 10.1007/s40472-016-0115-8

**Published:** 2016-10-07

**Authors:** Dominic A. Boardman, Jacinta Jacob, Lesley A. Smyth, Giovanna Lombardi, Robert I. Lechler

**Affiliations:** 1 MRC Centre for Transplantation, King’s College London, Guy’s Hospital, London, SE1 9RT UK; 2 NIHR Biomedical Research Centre, Guy’s & St Thomas’ NHS Foundation Trust & King’s College London, Guy’s Hospital, London, SE1 9RT UK; 3 School of Health, Sport and Bioscience, Stratford Campus, University of East London, London, E15 4LZ UK

**Keywords:** Transplantation, Allospecificity, Allorecognition, Multiple binary complexes, High determinant density

## Abstract

Direct allorecognition is the process by which donor-derived major histocompatibility complex (MHC)-peptide complexes, typically presented by donor-derived ‘passenger’ dendritic cells, are recognised directly by recipient T cells. In this review, we discuss the two principle theories which have been proposed to explain why individuals possess a high-precursor frequency of T cells with direct allospecificity and how self-restricted T cells recognise allogeneic MHC-peptide complexes. These theories, both of which are supported by functional and structural data, suggest that T cells recognising allogeneic MHC-peptide complexes focus either on the allopeptides bound to the allo-MHC molecules or the allo-MHC molecules themselves. We discuss how direct alloimmune responses may be sustained long term, the consequences of this for graft outcome and highlight novel strategies which are currently being investigated as a potential means of reducing rejection mediated through this pathway.

## Introduction

The ability of immune cells to distinguish between ‘self’ and ‘non-self’ is of fundamental importance. It ensures that invading pathogens are efficiently removed whilst tolerance towards cells of self-origin is maintained. In transplantation, the introduction of ‘non-self’ cells or tissues into a recipient can trigger an immune response. This is initiated when antigens derived from a genetically distinguishable member of the same species are recognised as foreign, a process termed ‘allorecognition’. Subsequent immune cell activation and elicitation of an immune response directed towards alloantigen-expressing cells ultimately results in graft-versus-host disease (GvHD) following bone marrow transplantation (BMT) or graft rejection following solid organ transplantation.

Organ transplantation is inherently an invasive surgical approach which is inevitably accompanied by ischemia/reperfusion injury, inflammation and tissue damage [1]. Consequently, innate immune responses such as the complement cascade [2, 3] are initiated which contribute to graft rejection [4]. However, studies conducted in neonatally thymectomised [5] and irradiated adult [6–8] mice have demonstrated that the most deleterious immune responses are driven by recipient-derived T cells. These cells have been described to recognise alloantigens via three pathways of allorecognition: the direct, indirect and semi-direct pathways. The direct pathway is initiated by donor-derived antigen-presenting cells (APC) which present allogeneic major histocompatibility complex (MHC)-peptide complexes to recipient T cells. Conversely, the indirect pathway relies on recipient-derived APCs which uptake, process and present allopeptides in the context of self-MHC class II. More recently, the semi-direct pathway was described in which recipient-derived APCs present both acquired, intact allo-MHC-peptide complexes (direct) and allopeptides in the context of self-MHC (indirect). In this review, we focus on the direct and semi-direct pathways of allorecognition.

### Premise of Direct Allorecognition

The unusual strength and vigour of direct alloimmune responses was first demonstrated by Bain et al. [9] through the use of in vitro-mixed leukocyte reactions (MLR). It was discovered that mixing leukocytes from two genetically unique individuals resulted in significant leukocyte activation, a phenomenon which was not observed by mixing leukocytes from genetically identical individuals. Subsequent in vivo studies demonstrated that similarly aggressive immune responses were observed in rodents which received allogeneic transplants [10]. This vigorous response was attributed to the presence of donor-derived ‘passenger’ leukocytes which were co-transferred into the recipient during the transplant procedure. Depletion of these cells from thyroid [11] or pancreatic [12] allografts, achieved by culturing the allografts in vitro to facilitate passenger leukocyte egression, resulted in a prolonged graft survival. In the former study, this prolongation was reversed by the infusion of donor peritoneal exudate cells (PEC), suggesting that recipient T cells with direct allospecificity must be activated by donor-derived APCs in order to destroy transplanted allografts [11].

Subsequent investigations performed by Lechler and Batchelor [13, 14] demonstrated that the principle ‘passenger’ leukocytes responsible for activating recipient T cells were dendritic cells (DC). In these studies, rat kidneys were ‘parked’ in intermediate recipients to deplete passenger leukocytes, prior to engraftment in a terminal recipient. The outcome was prolonged allograft survival which was prevented by the repletion of donor DCs, implicating a significant role for these cells in acute allograft rejection. Additional studies proceeded to suggest that these DCs prime and activate recipient T cells in secondary lymphoid tissues [15, 16].

### Models Explaining Direct Allorecognition

The strength and vigour with which direct alloimmune responses are elicited may be explained by the fact that all individuals have a high-precursor frequency of T cells specific for allogeneic MHC-peptide complexes. Approximately 0.01 % of the cells in a standard T cell repertoire are capable of responding to a specific foreign peptide presented by a self-MHC molecule. However, 1–10 % of these T cells can engage intact foreign MHC-peptide complexes (direct allorecognition) [17•]. Two models have been proposed to account for this unusually high frequency of T cells with direct allospecificity, each of which places an emphasis on the different components which comprise an MHC-peptide complex: the allopeptide and the allo-MHC molecule.

#### Peptide-Centric Model

The first model focuses on the contribution of the allopeptide bound in the groove of the allo-MHC. It is believed that specific structural components of self-MHC molecules are ‘mimicked’ by allo-MHC molecules. As such, self-restricted T cells dock and make contacts with allo-MHC molecules in the same manner as they would with self-MHC molecules. However, the binding groove of the self- and allo-MHC molecules is vastly different, thus the peptides presented by each is significantly different, despite being derived from similar endogenous proteins.

**Fig. 1:**
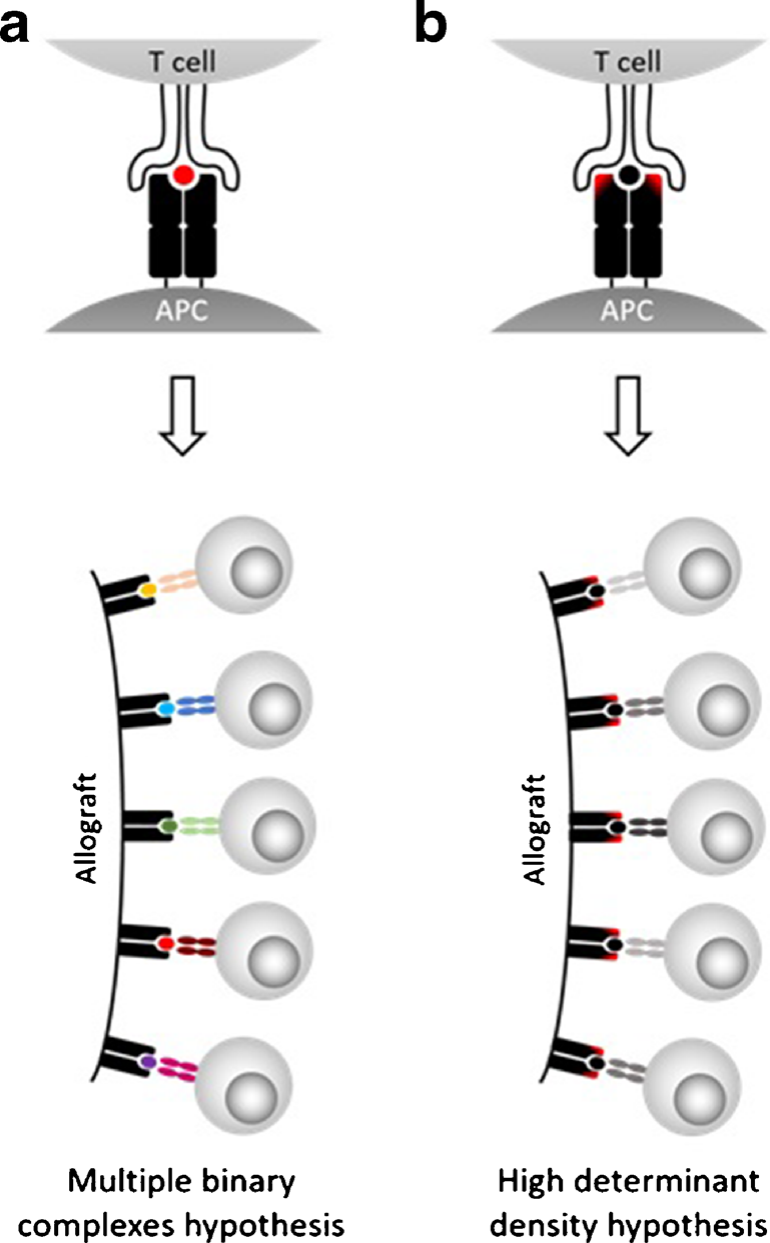
Comparison of the two principle theories explaining the high frequency of T cells with direct allospecificity. **a** Multiple binary complexes hypothesis. The elements of the allogeneic MHC molecule which interact with the TCR mimic those which are found in self-MHC molecules. As such, it is the presence of the allopeptide (*red*) which drives recognition of the allogeneic MHC-peptide complex. Allograft presentation of various allopeptides in the contexts of MHC molecules which are perceived as ‘self’ results in the activation of a range of T cells, each expressing a TCR specific for a different MHC-peptide complex. **b** High determinant density hypothesis. Structural differences in the polymorphic regions of the allo-MHC molecule are detected by the TCR (*red*). The high density of cognate allo-MHC molecules which possess these polymorphisms on donor-derived APCs facilitates the efficient activation of recipient T cells which recognise the allogeneic MHC molecule with a low, medium or high affinity

Given the random nature with which TCRs are genetically rearranged, a standard repertoire comprises T cells with a wide spectrum of specificities. As such, the recognition frequency of a self-MHC presenting a specific foreign peptide is low: often, a very small proportion of T cells engage a specific MHC-peptide complex. However, in this model, it is not one foreign peptide which is presented by allo-MHC molecules but instead, an entire pool of foreign peptides thus donor-derived cells will activate a variety of recipient T cells with a range of specificities. This hypothesis was initially proposed by Matzinger and Bevan in 1977 and is termed the ‘multiple binary complexes’ hypothesis (Fig. 1a) [18].

In 1988, Eckels et al. [19] demonstrated the importance of allopeptides in the activation of T cells with direct allospecificity. In this study, HLA-DR1-restricted alloreactive T cell clones were co-cultured with allogeneic APCs in the presence and absence of a competing peptide. T cell proliferation induced by the presentation of allopeptides in the context of HLA-DR1 was abrogated when the allopeptides were displaced by competing influenza haemagglutinin-based peptides. Subsequently, Panina-Bordignon et al. [20] demonstrated that APC presentation of peptides derived from endogenous proteins contributes significantly to the activation of alloreactive T cell clones. Of 1489 CD4^+^ T cell clones analysed, 6.6 % specifically responded to APCs which presented human serum albumin (HSA)-derived peptides but not foetal calf serum (FCS)-derived peptides, despite the peptides being presented by the same HLA-DR molecule. Conversely, it has been shown that presentation of allo-MHC molecules lacking allopeptides, achieved through the use of MHC mutants [21] or acid-treatment of target cells [22], triggers a limited response from alloreactive T cells. In the latter study, T cell responses were restored by the addition of synthetic allopeptides. Furthermore, various groups have described allopeptide sequence consensuses [23] and have demonstrated that through disrupting the binding of these specific allopeptides, there is a loss of response from alloreactive T cells [24]. More recent studies have employed point mutation approaches to determine which motifs in a TCR are important for eliciting T cell activation and have concluded that TCR-peptide interactions are fundamental in direct allorecognition [25].

#### MHC-Centric Model

The second model focuses on the fact that allo-MHCs are structurally different to self-MHCs. Whilst the majority of elements recognised by a TCR are conserved across various MHC subtypes [26], specific amino acid polymorphisms present in the allo-MHC molecule may modify the manner by which a self-restricted TCR docks with an MHC, irrespective of the peptide presented.

In this model, it is suggested that fundamental differences exist between self- and allo-MHCs in specific polymorphic residues which are exposed to potential docking TCRs. These residues cause the MHC-peptide complex to be recognised as foreign, thus the peptide presented stabilises the MHC-peptide complex but has little influence in the recognition process. Additionally, the affinity with which this TCR:allo-MHC interaction occurs may have implications in the alloresponse observed. Whilst T cells are selected to bind self-MHC-peptide complexes with a low affinity, it is possible that they would bind allo-MHC-peptide complexes with a high affinity, suggesting that a high affinity cross-reaction is responsible for the allorecognition observed. Furthermore, the high density of ligands expressed by donor APCs can further facilitate the activation of alloreactive T cells. This hypothesis is termed the ‘high determinant density’ hypothesis and was originally proposed by Bevan in 1984 (Fig. 1b) [27].

In 1989, Schneck et al. [28] developed a peptide which mimicked a specific region of the MHC class I molecule H-2K^b^. In the presence of this peptide, the cytotoxic activity of H-2K^b^-specific CD8^+^ T cells against H-2K^b^ target cells was inhibited, demonstrating the importance of the TCR:MHC interaction in this alloresponse. Subsequently, and in contrast to the results of Wang et al. [22], Smith et al. demonstrated in a mouse setting that removal of peptides bound to MHC molecules through the use of acid washing did not perturb the ability of T cells to bind and react to allo-MHC molecules [29]. Lombardi et al. [30] proceeded to present corresponding findings in a human T cell setting. In this study, a site-directed mutagenesis approach was employed to generate genetically altered HLA-DR molecules which were transfected into murine DAP.3 cells. Using these artificial APCs, it was demonstrated that specific mutagenesis of TCR contact regions in the MHC molecule resulted in inhibition of T cell binding and subsequent effector responses. As such, it was evident that specific sites of the allo-MHC molecule were critical for direct allorecognition to occur. These findings were later confirmed by Villadangos et al. who employed a similar approach whilst mutating HLA-B27 [31].

### Conundrum of Allorecognition

T cell progenitors must undergo a stepwise ‘education’ process in the thymus to develop into mature T cells. Thymocytes expressing a TCR capable of recognising peptides presented in the context of self-MHC molecules are ‘positively selected’, whilst thymocytes that recognise self-MHC-peptide complexes with a high affinity are ‘negatively selected’. As such, the mature T cells which results from this process are able to recognise self-MHC-peptide complexes with a low affinity.

The existence of this process reveals a conundrum: why do self-restricted T cells [32] recognise allo-MHC-peptide complexes? Studies have demonstrated that cross-reactivity between self and allogeneic MHC-peptide complexes is the key for this mode of allorecognition. In other words, T cells specific for peptide ‘x’ presented by self-MHC ‘A’ are also able to recognise peptide ‘y’ presented by allo-MHC ‘B’ [33]. Studies supporting this have demonstrated that LFA-3^+^ [34] and CD45RO^+^ [35] memory T cells primed against peptide ‘x’ presented by self-MHC ‘A’ also respond to allo-MHC-peptide complexes (peptide ‘y’ presented by allo-MHC ‘B’). Furthermore, these cross-reacting memory T cells comprise a significant proportion of the total T cells which respond in a direct manner. This cross-reactivity concept was further accentuated by Lombardi et al. in 1989 [36]. In this study, human alloreactive T cell clones which were specific for HLA-DR1 were co-cultured with autologous APCs presenting *Candida albicans*-derived antigens in the context of HLA-DR4/HLA-DR13. Half of the alloreactive T cell clones analysed responded in these co-cultures, suggesting that cells which were capable of recognising the allo-MHC molecule HLA-DR1 had previously been activated by APCs presenting self-MHC-peptide complexes.

### Structural Importance of TCRs and MHCs

The hypotheses and accompanying functional studies described have suggested that the attention of T cells with direct allospecificity is either for the allopeptides or the allo-MHC molecules. However, further insight into the molecular mechanisms of these interactions was provided by structural studies which employed x-ray crystallography. In 1987, Bjorkman et al. first published the structure of HLA-A2 [37]. Subsequent studies performed with HLA-A2 [38] and HLA-B27 [39] suggested specific residues which were important for allorecognition. However, significant progress in this field was not made until 1996 when co-crystals containing a TCR interacting with an MHC-peptide complex were generated [40, 41]. These studies confirmed for the first time that TCRs bind MHC-peptide complexes in a diagonal orientation, demonstrating that both the MHC and the peptide are recognised. Specifically, the variable Vα domain of the TCR positions above the N-terminal half of the peptide and the Vβ domain locates above the C-terminal half of the peptide [42, 43]. This places an emphasis on the complementarity-determining region (CDR)3 loops of the TCR [44], the most variable region of the TCR, for recognising the peptide whilst the CDR1 and CDR2 loops primarily interact with the MHC.

These studies accentuate the plasticity with which a TCR is able to cross-react and engage a variety of different MHC-peptide complexes [45, 46]. Indeed, this TCR degeneracy (ability of a single TCR to engage multiple MHC-peptide complexes) has fundamental ramifications in that it allows a finite number of T cells to recognise a potentially infinite number of MHC-peptide complexes [46, 47]. Although various theories exist to explain this degeneracy [48], it is clear that the ability of a TCR to change conformation upon engagement of a cognate MHC-peptide complex is paramount [42].

In 2007, Colf et al. [49] demonstrated that whilst a single TCR could cross-react to recognise self-MHC and allo-MHC molecules, different TCR conformations were required to accomplish this. The structure of the mouse-based TCR ‘2C’ was compared when engaged with the self-MHC-peptide complex H2K^b^-dEV8 and the allo-MHC-peptide complex H2L^d^-QL9 [50]. Genetic manipulation of the CDR3α to yield a high-affinity variant of the 2C TCR did not influence the orientation with which the allo-MHC-peptide complex H2L^d^-QL9 was bound. As such, interrupting the CDR3α-peptide interaction had little effect on the binding of the TCR to the allo-MHC-peptide complex, suggesting an MHC-centric model may be prevalent in this setting [50].

Conversely, structural data has also suggested a crucial role for allopeptides in driving direct allorecognition. Studies by Reiser et al. [44, 51, 52] conducted using mouse-based TCRs have detailed key conformational changes which occur in the CDR3 loops of a TCR upon binding of an allogeneic MHC class I molecule. Here, the CDR3α loop of the TCR ‘BM3.3’ was found to adopt different conformations depending on the peptide presented: presentation of the ‘pBM1’ peptide caused the CDR3α loop to fold away from the peptide binding groove [52] but when the ‘VSV8’ peptide was presented, the CDR3α loop pointed towards the amino-terminal end of the peptide [51]. Furthermore, given the fact that relatively minor changes were observed in the CDR1 and CDR2 loops, these results suggest that the allopeptide is responsible for driving direct allorecognition [48, 53]. Studies conducted with human TCRs interacting with HLA-B molecules have also yielded results in favour of a peptide-dependent direct allorecognition model. It has been suggested that MHC molecular mimicry is the basis for the cross-reactivity observed between self-HLA-B*0801 and allo-HLA-B*3501 [54] as well as self-HLA-B*0801 and allo-HLA-B*4402/4405 [55]. In the latter study, the nature with which the TCR ‘LC13’ interacted with self-HLA-B*0801 and allo-HLA-B*4405 was remarkably similar; a comparable number of van der Waals interactions, hydrogen bonds and salt-bridges were formed in each case. Additionally, despite the fact that drastically different peptides were presented, very similar contacts were made by the CDR loops interacting with the self- and allo-HLA-B molecules.

Overall, the aforementioned high determinant density and multiple binary complex models provide two explanations for why T cells with direct allospecificity exist with a high-precursor frequency. With functional and structural data supporting both hypotheses, it is likely that in vivo, the high frequency of direct allorecognition can be attributed to a combination of these theories.

### Consequences of Direct Allorecognition

Allorecognition typically leads to an effector response in which CD8^+^ T cells with direct allospecificity actively kill donor-derived target cells [56], leading to allograft dysfunction and failure. Various studies have investigated how recipient graft-specific CD8^+^ and CD4^+^ T cells contribute to acute and chronic transplant rejection [33, 57–60]. For example, in 2000, Pietra et al. [61] investigated the contribution of CD4^+^ T cells in acute graft rejection through the use of severe combined immunodeficiency (SCID) and recombination-activating gene (RAG)-1 deficient mice which lack functional T and B cells. C57BL/6 heart allografts, which survived indefinitely in SCID mice, were acutely rejected (mean survival time (MST) of 12 days) when BALB/c CD4^+^ T cells were adoptively transferred on the day of transplant. Conversely, heart allografts which lacked donor C57BL/6 MHC class II molecules (C2D donor mice) were not rejected (7/8 allografts survived >60 days), demonstrating that CD4^+^ T cells directly specific for donor MHC class II molecules were necessary for acute allograft rejection.

More recently, Brown et al. [62] further demonstrated the contribution of recipient CD4^+^ T cells to allograft rejection using a fully mismatched kidney transplant model in which donor APCs were specifically depleted. In this study, one native kidney of recipient (FVB) mice was replaced with an allogeneic (C57BL/6 × CBA F1) kidney. In the absence of treatment, transplanted kidneys were rejected acutely in 40 % of cases. However, in recipients which were treated with an immunotoxin-conjugated antibody specific for donor MHC class II (I-A^k^) to depleted donor APCs, kidney allografts were completely protected: histological analysis showed no evidence of rejection and upon removal of the second native kidney, the function of the transplanted kidney (blood urea nitrogen score) was found to be intact.

Analysis of blood samples acquired from stable renal transplant recipients has revealed that recipient CD4^+^ T cells with direct allospecificity become hyporesponsive towards alloantigens and are not deleted [63]. This work was further extended by demonstrating that that human CD4^+^ T cells co-cultured with MHC class II-expressing thyroid follicular cells (TFC) [64] or epithelial cells [65] do not proliferate or produce cytokines in the absence of co-stimulation and are hyporesponsive upon subsequent challenge with EBV-transformed lymphoblastoid B cell lines (B-LCL) [66]. Together, these results suggest that in the absence of donor-derived professional APCs, recipient CD4^+^ T cells engage MHC class II molecules presented by transplanted tissue parenchymal cells which lack co-stimulatory molecules. As a result, these T cells become anergic [65] or polarised towards a Th2 phenotype [64], suggesting that prolonged alloimmune responses depend on an alternative mode of allorecognition.

### Sustaining a Long-Term Direct Alloimmune Response

During a transplant procedure, donor APCs are transferred but these cells are killed or die over time [56]. As such, it has historically been believed that the direct pathway of allorecognition predominates during acute graft rejection. Furthermore, as previously discussed, in the absence of donor APCs, T cells with direct allospecificity recognise allo-MHC presented on the allograft parenchyma which leads to anergy induction [66].

As previously discussed, Lechler and Batchelor [13, 14] demonstrated that prolonged rat kidney allograft survival could be achieved by ‘parking’ the allograft in an intermediate recipient to deplete passenger leucocytes. However, the fact that these allografts were eventually rejected led to the proposal of the indirect pathway of allorecognition whereby recipient-derived APCs consistently sample and present donor antigens provided by the allograft. As such, the indirect pathway has been believed to be the main mode of chronic rejection [67].

As antigens acquired from exogenous origins are naturally presented in the context of MHC class II, it is primarily CD4^+^ T cells which recognise alloantigens in an indirect manner, not CD8^+^ T cells which are responsible for eliciting cytotoxicity. Furthermore, efficient CD8^+^ T cell activation requires help from CD4^+^ T cells [60]. As such, it is reasonable to conclude that a link between the direct and indirect pathways of allorecognition exists and that CD4^+^ T cells with indirect allospecificity facilitate the activation of CD8^+^ T cells with direct allospecificity [59, 68]. Theoretically, for this to occur, two separate APCs should be present: a recipient APC presenting allopeptides indirectly to the CD4^+^ T cell and a donor APC presenting antigen directly to the CD8^+^ T cell. However, from a practical viewpoint, it is highly unlikely that these two APC:T cell interactions naturally occur in such close proximity, leading to a conundrum termed the ‘four-cell conundrum’. This was resolved by the discovery of APCs which present intact donor MHC-peptide complexes in a direct manner and allopeptides in an indirect manner [69–71], the basis of a more recently described third pathway of allorecognition: the semi-direct pathway [70].

In a similar manner to the indirect pathway, the semi-direct pathway relies on recipient-derived APCs which infiltrate the allograft following engraftment. However, in addition to presenting allopeptides indirectly, these cells acquire intact donor MHC-peptide complexes from donor-derived cells/tissues, a phenomenon termed ‘cross-dressing’, thus present allogeneic MHC-peptide complexes in a direct manner [71]. Indeed, DCs presenting both donor MHC class I-peptide complexes (direct presentation) and allopeptides in the context of self-MHC (indirect presentation) have been observed following skin [72••], kidney [73] and heart [74, 75••] transplantation. Through this pathway, direct alloimmune responses can continue long after donor-derived APCs have died, but the extent to which this pathway contributes to allograft rejection is not yet known. Recently, we have shown that after removal of the direct pathway and in the absence of cross-presentation, acquired allo-MHC-peptide complexes, on recipient DCs, can drive allograft rejection throughout the life-span of the transplant [72].

### Targeting T cells with Direct Allospecificity

Over the past few decades, advances in surgical techniques and the development of modern immunosuppressive regimens have enabled transplantation of cells/organs to become a viable treatment option for a plethora of different conditions. Although the mechanisms by which these immunosuppressive drugs function are not completely understood, it is believed their benefits stem through the suppression of T cells with direct allospecificity [76]. However, drug-based immunosuppression is inherently non-specific and associated with undesirable side-effects, leaving recipients under consistent nephrotoxic insult with an increased susceptibility to acquiring infections and developing cancer [76]. As such, various strategies are currently under investigation to reduce the requirement for these drugs.

Following transplantation, recipient T cells with direct allospecificity are initially activated by ‘passenger leukocytes’. Given the deleterious consequences of these cells, strategies have been explored to investigate whether depletion of these cells from allografts can reduce the intensity of an initial immune insult. Brown et al. [62] recently achieved this through the use of a donor MHC class II-specific immunotoxin-conjugated antibody. As described above, these authors observed indefinite kidney allograft survival and function in mice which received this treatment. Additionally, Stone et al. [77•] have demonstrated that the proportion of passenger leukocytes in lung allografts can be significantly reduced through the use of ex vivo lung perfusion (EVLP). These authors employed a model whereby donor pig lungs are explanted, perfused ex vivo and then transplanted into recipient pigs. EVLP, which did not severely alter the viability of the graft, reduced both donor leukocyte egression and recipient T cell infiltration post-transplantation, suggesting a potential clinical benefit of passenger leukocyte removal prior to allograft implantation.

Luo et al. have explored the possibility of inducing donor-specific transplant tolerance by infusing ethylene carbodiimide (ECDI)-fixed donor-derived APCs before and after a transplant procedure [78]. Fuelled by similar approaches which were applied to treat multiple sclerosis [79] and diabetes [80] in mice, the authors of this study demonstrated that indefinite survival (>100 days) of fully mismatched islets could be achieved by infusing recipient mice intravenously with 100 million donor-derived ECDI-treated splenocytes before and after the islet transplant procedure. Furthermore, it was demonstrated that CD4^+^CD25^+^ Tregs had a crucial role in tolerance induction. These authors have also demonstrated that similar levels of tolerance can be achieved through the use of biodegradable particles (poly (lactide-co-glycolide); PLG) which present donor antigens (PLG-dAg) as a substitute for the aforementioned ECDI-treated splenocytes [81].

As suggested, Tregs play a fundamental role in inducing tolerance in vivo. Allograft survival is significantly prolonged by increasing the proportion of Tregs in recipient mice, either by promoting endogenous Treg expansion [82] or adoptively transferring ex vivo-expanded Tregs [83, 84]. Similarly, observational studies performed on human samples have noted a correlation between the proportion of Tregs and allograft survival [85, 86]. These studies paved the way for phase I/II clinical trials which are currently investigating the safety and efficacy of polyclonal Treg therapy in kidney (The ONE Study), liver (ThRIL) and bone marrow transplant recipients [87]. Furthermore, we [88•, 89] and others [90] have demonstrated that Tregs with direct allospecificity are superior to polyclonal Tregs at prolonging allograft survival in vivo. In these studies, human Tregs with direct allospecificity were preferentially expanded using allogeneic DCs [88•] or B cells [89, 90]. Using human skin xenograft transplant models, direct allospecific Tregs were shown to inhibit direct alloimmune-mediated skin injury significantly more effectively than polyclonal Tregs. However, we have also observed in a mouse setting that Tregs with direct allospecificity alone are insufficient to prolong the survival of heart allografts [91]. To achieve this, Tregs required both direct and indirect allospecificity, suggesting that it is necessary to block both direct and indirect allospecific Teffs in order to reduce allograft damage in this setting. Given the superior efficacy of Tregs with direct allospecificity, compared to polyclonal Tregs, the safety and efficacy of these cells is currently being assessed clinically in kidney (DART as part of The ONE Study: NCT02244801) and liver (deLTa: NCT02188719 and NCT01624077) transplant recipients.

## Conclusions

The mechanisms of direct allorecognition have puzzled immunologists for decades. Why self-restricted T cells recognise allo-MHC-peptide complexes and with a high-precursor frequency remain a mystery. Theories proposed to offer an explanation for these conundrums attribute the phenomenon of direct allorecognition to either the presence of allopeptides or allo-MHC molecules. Both theories are supported by functional and structural data, suggesting that in vivo, both allopeptides and allo-MHC molecules are responsible for driving direct allorecognition. For many decades, the direct pathway of allorecognition was believed to be solely responsible for early alloimmune-mediated rejection. However, the more recent discovery of the semi-direct pathway by our group has demonstrated how rejection mediated by T cells with direct allospecificity can be sustained long-term. As such, various strategies are currently being explored as potential means of limiting direct allorecognition and inducing tolerance.
